# Effect of *KRAS* codon13 mutations in patients with advanced colorectal cancer (advanced CRC) under oxaliplatin containing chemotherapy. Results from a translational study of the AIO colorectal study group

**DOI:** 10.1186/1471-2407-12-349

**Published:** 2012-08-09

**Authors:** Anke Reinacher-Schick, Karsten Schulmann, Dominik P Modest, Nina Bruns, Ulrich Graeven, Malgorzata Jaworska, Richard Greil, Rainer Porschen, Dirk Arnold, Wolff Schmiegel, Andrea Tannapfel

**Affiliations:** 1 Department of Internal Medicine, Knappschaftskrankenhaus, Ruhr-University Bochum, Bochum, Germany; 2 Department of Internal Medicine III, Klinikum der Universität, München, Germany; 3 Department of Hematology, Oncology and Gastroenterology, Kliniken Maria Hilf, Mönchengladbach, Germany; 4 Institute of Pathology, Ruhr-University Bochum, Bochum, Germany; 5 3rd Medical Department with Hematology and Medical Oncology, Oncologic Centre Paracelsus Medical University, Salzburg, Austria; 6 Klinikum Bremen-Ost, Bremen, Germany; 7 Hubertus Wald Cancer Center, University Hospital Hamburg, Hamburg, Germany; 8 Department of Gastroenterology & Hepatology, Berufsgenossenschaftliches Klinikum Bergmannsheil, Ruhr-University Bochum, Bochum, Germany; 9 Center for Clinical Studies in Oncology within PURE, Ruhr-University Bochum, Bochum, Germany; 10 Medical Department, Knappschaftskrankenhaus, Ruhr-University Bochum, In der Schornau 23-25, Bochum, 44892, Germany

**Keywords:** Codon 13 mutation, Colorectal cancer, *KRAS*, Oxaliplatin, Prognosis

## Abstract

**Background:**

To evaluate the value of *KRAS* codon 13 mutations in patients with advanced colorectal cancer (advanced CRC) treated with oxaliplatin and fluoropyrimidines.

**Methods:**

Tumor specimens from 201 patients with advanced CRC from a randomized, phase III trial comparing oxaliplatin/5-FU vs. oxaliplatin/capecitabine were retrospectively analyzed for *KRAS* mutations. Mutation data were correlated to response data (Overall response rate, ORR), progression-free survival (PFS) and overall survival (OS).

**Results:**

201 patients were analysed for *KRAS* mutation (61.2% males; mean age 64.2 ± 8.6 years). *KRAS* mutations were identified in 36.3% of tumors (28.8% in codon 12, 7.4% in codon 13). The ORR in codon 13 patients compared to codon 12 and wild type patients was significantly lower (p = 0.008). There was a tendency for a better overall survival in *KRAS* wild type patients compared to mutants (p = 0.085). PFS in all patients was not different in the three *KRAS* genetic groups (p = 0.72). However, we found a marked difference in PFS between patients with codon 12 and 13 mutant tumors treated with infusional 5-FU versus capecitabine based regimens.

**Conclusions:**

Our data suggest that the type of *KRAS* mutation may be of clinical relevance under oxaliplatin combination chemotherapies without the addition of monoclonal antibodies in particular when overall response rates are important.

**Trial registration number:**

2002-04-017

## Background

The oncogene KRAS belongs to the protein family of small G-proteins and is mutated in 35-40% of colorectal cancers (CRC) [1]. *RAS* mutations are considered early events in colon carcinogenesis and are well conserved between primary tumor and corresponding metastases [2]. *KRAS* mutations tested routinely include six mutations in codon 12 and one mutation in codon 13.

*KRAS* has been studied extensively as a prognostic marker in CRC, but results are still conflicting. Overall, there seems a tendency towards inferior outcome for patients with *KRAS* mutant tumors even in large randomized trials [3-7]. Importantly, *KRAS* has recently been identified as a strong predictive marker for patients with advanced CRC under anti-EGFR-treatment. Various studies demonstrated that while patients with tumors expressing wild type KRAS may benefit from anti-EGFR (epidermal growth factor receptor)-antibody treatment, patients carrying a mutated *KRAS* gene do not [5,8-10]. In contrast, two large trials using oxaliplatin-based chemotherapy-backbones did not confirm *KRAS* wild type to be a powerful predictor of treatment efficacy in metastatic CRC [4,11]. However, the studies published until recently did not differentiate between the different *KRAS* mutations and other histopathological or molecular features of their patients. The clinical observation, that some patients with *KRAS* mutation may respond to anti-EGFR-antibody therapy, as well as experimental data, that the biological effects of *KRAS* mutations may differ, was addressed recently by de Roock and Tejpar [12,13]. They reported that patients with codon 13 mutations in *KRAS* exhibit a worse overall prognosis with short overall survival times under standard chemotherapy, but may benefit from anti-EGFR-antibody therapy similar to wild type patients. These observations prompted us to look for the effect of the *KRAS* mutational status in correlation with response and survival data in patients with advanced colorectal cancer receiving oxaliplatin containing chemotherapy from a prospective randomized multicenter phase III trial of the German AIO study group.

## Methods

### Patients

All patients participated in a prospective randomized phase III first-line palliative chemotherapy trial of advanced CRC of the AIO colorectal study group (Arbeitsgemeinschaft Internistische Onkologie of the German Cancer Society). The study was performed according to the Helsinki declaration and it was approved by the ethics review board of the Central Hospital Bremen and of the Medical Faculty of the Ruhr-University Bochum. The study design, patient characteristics, treatment plans and results of the clinical trial have been reported previously [14]. Briefly, a total of 474 patients were randomized to be treated with either 5-FU/folinic acid (FA) and oxaliplatin (FUFOX: oxaliplatin, 50 mg/m^2^; FA, 500 mg/m^2^; continuous 5-FU, 2,000 mg/m^2^/22 h; on day 1, 8, 15, 22; q day 36) or capecitabine and oxaliplatin (CAPOX: oxaliplatin, 70 mg/m^2^ on day 1 and 8; capecitabine, 2 × 1,000 mg/m^2^/day consecutively for 2 weeks, q day 22). No clinical factor was found to be predictive for definition of a subgroup of patients benefiting more or less from each fluoropyrimidine backbone. We here present data on a subcohort of 201 patients (42.4%) with available formalin-fixed paraffin-embedded tissue. Samples were retrieved from pathologists responsible for first diagnosis, pseudononymized and forwarded to the Institute of Pathology of the Ruhr-University Bochum, which was blinded to treatment allocation and prognostic outcomes.

### DNA extraction and mutation analysis

DNA was extracted from anonymized formalin-fixed paraffin embedded tissue samples. For each patient, five 10-μm sections were prepared. An additional representative 1-μm section was deparaffinized, stained with H&E, and analyzed for detailed morphology. Regions displaying tumor cellularity of >70% were marked and macrodissected. Tissue was extracted using QIAmp DNA Mini kit (Qiagen, Hilden, Germany). Real-Time PCR amplification for the most frequent seven *KRAS* mutations was performed using commercially available kits from DxS Ltd. (Manchester, UK) according to the manufacturer’s instructions. This kit detects >95% of known *KRAS* mutations. Laboratory staff was blinded to the patient data and clinical outcome. *BRAF* mutation analysis to detect the V600E substitution was performed by RT-PCR (see supplemental file for further details).

### Statistical analysis

Chi-square test was used to evaluate the associations between *KRAS* status and other dichotomous variables. The analysis of progression-free survival (PFS) and overall survival (OS) was done using the Kaplan-Meier method and differences between subgroups were calculated by log-rank test. Data have been analyzed using SPSS 18.0 (Munich, Germany). All tests were two sided. For all tests, *p* values <0.05 were considered significant.

## Results

### Patient characteristics

The subcohort of patients successfully analyzed for *KRAS* mutations consisted of 201 patients (61.2% males with a mean age of 64.2 ± 8.6 years, range 35 – 82). Colon cancer was diagnosed in 131 (65.2%) patients while rectal cancer was diagnosed as primary tumor in 61 (30.3%) patients. Localization was not known for 9 cases (4.5%). 105 (52.2%) patients were treated with FUFOX, whereas 96 (47.7%) patients received CAPOX. 115 (57.2%) patients had synchronous metastases, whereas 59 (29.4%) had metachronous metastases. Whether metastases were synchronous or metachronous was not known for 27 ( 13.4%) patients (Table1). The subcohort reported here was representative of the complete study cohort with respect to age, gender, treatment plans (i.e. percentage of patients with FUFOX and CAPOX respectively). Median PFS and OS of the total ITT (intention-to-treat) population [14] and the biomarker subpopulation were fully comparable (PFS under FUFOX was 8.0 in the ITT cohort vs. 7.8 in the *KRAS* cohort; PFS under CAPOX was 7.1 vs. 7.0; OS under FUFOX was 18.8 in the ITT cohort vs. 17.5 in the *KRAS* subcohort and OS under CAPOX was 16.8 vs. 18.4).

**Table 1:** Baseline characteristics of the investigated subcohort

**Biomarker population**	**N**	**%**
**Age**		
**Mean 64.15 years**		
**Range 35–82 years**		
**≤60 y**	58	28.9
**>60 y**	143	71.1
**Sex**		
**Female**	78	38.8
**Male**	123	61.2
**Treatment arm**		
**FUFOX**	105	52.2
**CAPOX**	96	47.8
**Localization of primary tumor**		
**Colon**	131	65.2
**Rectum**	61	30.3
**n.k.**	9	4,5
**T stage at initial diagnosis**		
**T1**	1	0.4
**T2**	15	7.5
**T3**	132	65.7
**T4**	49	24.4
**n.k.**	4	2.0
**N stage at initial diagnosis**		
**N0**	46	22.9
**N1**	57	28.3
**N2**	89	44.3
**n.k.**	9	4.5
**M stage at initial diagnosis**		
M0*	59	29.4
**M1**	115	57.2
**n.k.**	27	13.4

*M0 indicates metachronous metastatic disease at initial diagnosis of CRC, M1 indicates synchronous metastatic disease at initial diagnosis of CRC. *n.k.* = not known – there were no data available.

### Mutation frequency

*KRAS* mutations could be successfully analyzed in 201 samples. We identified *KRAS* mutations in 73/201 (36.3%) tumors. 58 (28.9%) of *KRAS* mutations were located in codon 12, whereas 15 (7.46%) were found in codon 13(see Table2). The three most frequent *KRAS* alterations in our samples were c.35 G > A (G12D, n = 25; 12.4%), c.38 G > A (G13D, n = 15; 7.46%), and c.35 G > T (G12V, n = 13, 6.46%). In 128 patients no *KRAS* mutation was found (63.6%). In these patients we detected 13 mutations in the *BRAF* gene (V600E) (10% of wild type patients).

**Table 2:** ** *KRAS* mutation frequency in the investigated subcohort ( *KRAS* Wild type: 128 (63.7%), *KRAS* Mutation 73 (36.3%))**

			**relative frequency of occurrence (%)**	**absolute frequency of occurrence**
**CODON 12 mutation:**				
Aspartate (G12D)		c.35G>A	12,4	25
Valine (G12V)		c.35G>T	6,5	13
Alanine (G12A)		c.35G>C	3,4	7
Cysteine (G12C)		c.34G>T	3	6
Serine (G12S)		c.34G>A	3	6
Arginine (G12R)		c.34G>C	0,5	1
**CODON 13 mutation:**				
Aspartate (G13D)		c.38G>A	7,5	15

### Correlation between mutations and response rate

Tumor response evaluation was available for 201 patients. Grouping all *KRAS* mutations together, mutated tumors were associated with a significantly lower response rate (RR; defined as partial or complete remission by RECIST) as compared to tumors without *KRAS* mutations (44.4% *vs.* 63.0%, p = 0.012). When patients with codon 13 mutated tumors were analysed separately the overall response rate in this cohort was 23% as compared to 49% in codon 12 mutated tumors and 63% in wild type tumors (Table3, p = 0.008). Disease control rates (DCR) were 77%, 81% and 88%, respectively, which was not statistically significant (Table3, p = 0.29).

**Table 3:** **Tumor response assessment and correlation to *KRAS* mutational status**

**All patients**	**All**	**WT**	**Codon 12 mutation**	**Codon 13 mutation**	**p-value**
**No. of patients%**	**201**	**128**	**58**	**15**	
	**100**	63.6	28.9	7.5	
**ORR%**	**55**	**63**	**49**	**23**	**0.008**
**95% CI**	**(49–61)**	(54–71)	(37–62)	(8–51)	(Chi-Square)
**DCR%**	**85**	**88**	**81**	**77**	**0.29**
**95% CI**	**(80–90)**	(80–92)	(68–89)	(49–93)	(Chi-Square)

All patients. Percentages based on non-missing data, p-values for WT vs Codon 12 mutations vs codon 13 mutations; *WT* wild type; *ORR* overall response rate, *DCR* disease control rate.

### Correlation between mutations and progression-free survival

**Figure 1:**
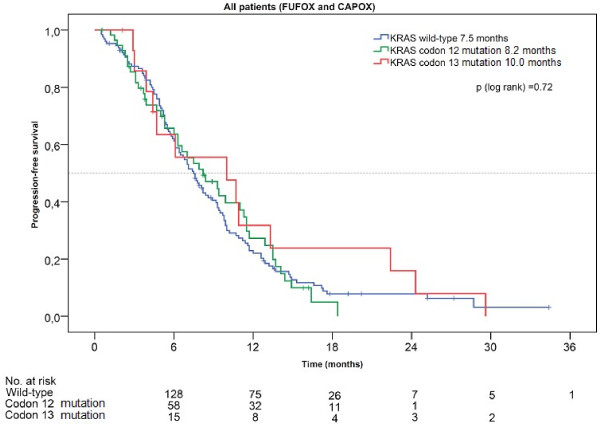
**Progression-free survival according to *KRAS* status.**

During follow-up, 170 of 201 evaluable patients had progressed. The median PFS in all patients of the *KRAS* subcohort was not statistically different in relation to the *KRAS* mutational status (wild type: 7.5 months, mutation codon 12: 8.2 months, mutation codon 13: 10.0 months; p = 0.71) (Figure1). However, when analysing the two treatment arms separately, we found a substantial, non-significant, difference in PFS in codon 13 mutated tumors versus codon 12 mutated tumors. While median PFS was as low as 6.1 months for codon 13 patients receiving infusional 5-FU, median PFS was 13.3 months in patients treated with capecitabine (HR: 2.52, p = 0.22). Patients with codon 12 mutations showed a trend towards the opposite effect: median PFS was 7.0 months under CAPOX therapy while median PFS was 9.9 months under FUFOX (HR: 0.62, p = 0.12) (Table4).

**Table 4:** **PFS (progression free survival) according to *KRAS* mutation for the two different treatment arms**

**PFS**	**FUFOX**	**CAPOX**	**p-value (log rank)**
	**n = 105**	**n = 96**	**HR (95% CI)**
**All patients**	**8.2 months**	**6.4 months**	**0.18** **0.81 (0.61-1.09)**
**WT**	**8.1**months	**6.4**months	**0.29**
			**0.82** (0.56-1.19)
**Codon 12 mutation**	**9.9**months	**7.0**months	**0.12**
			**0.62** (0.33-1.14)
**Codon 13 mutation**	**6.1**months	**13.3**months	**0.22**
			**2.52** (0.54-11.70)

PFS, *HR* hazard ratio by cox regression, *CI* confidence interval, *WT* wild type.

### Correlation between mutations and overall survival

**Figure 2:**
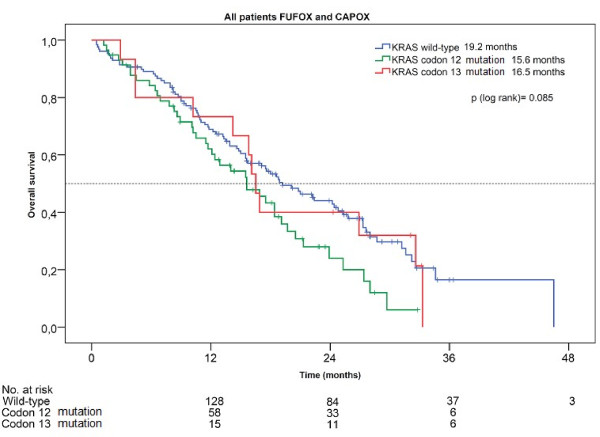
Overall survival according to KRAS status.

During follow-up 135 of 201 evaluated patients had died. We observed a trend towards a better survival time in patients with wild type tumors compared to those with a mutation of *KRAS*. The overall survival of wild type *KRAS* patients was 19.2 months, for patients with codon 12 mutations 15.6 months and for patients with codon 13 mutations 16.5 months. These differences were of marginal significance (p = 0.085) (Figure2).

Evaluating the different treatment arms separately, we found comparable survival times without significant differences (Table5). Since the survival curves seemed to separate for wild type *KRAS* and mutant *KRAS* patients at 14 months we hypothesised that this difference was most likely caused by the influence of post-study treatment. 71 out of 128 (55.4%) wild type patients received further lines of therapy while 32 out of 128 wild type patients (25%) received cetuximab. Out of 73 patients with *KRAS* mutations 42 were treated in further lines (57.5%) with 10 patients receiving cetuximab (13.7%). When analysing post progression survival (PPS) in these patients receiving second and further line therapies, patients receiving cetuximab had a significant better overall survival when compared to those patients under irinotecan therapy only, irrespective of the *KRAS* status. PPS in *KRAS* wild type patients: 32.2 months when cetuximab was given and 18.8 months when cetuximab was not included (p < 0.001). PPS for *KRAS* mutant patients: 27.9 months under cetuximab and 16.9 months without cetuximab (p = 0.032).

**Table 5:** **OS (overall survival) according to *KRAS* mutation for the two different treatment arms**

**OS**	**FUFOX**	**CAPOX**	**p-value (log rank)**
	**n = 105**	**n = 96**	**HR (95% CI)**
**WT**	**24.2**months	**18.9**months	**0.31**
			**0.79** (0.51-1.24)
**Codon 12**	**15.6**months	**15.5**months	**0.54**
**mutation**			**0.83** (0.45-1.53)
**Codon 13**	**16.1**months	**16.5**months	**0.62**
**mutation**			**1.39** (0.37-5.37)

OS, *HR* hazard ratio by cox regression, *CI* confidence interval.

## Discussion and conclusions

We assessed the prognostic value of *KRAS* codon 12 and codon 13 mutations in tumor tissue from patients with advanced CRC recruited into a phase III clinical trial using CAPOX or FUFOX treatment regimens. This is the first randomized phase III trial retrospectively investigating the role of codon 13 mutations in advanced CRC patients treated with oxaliplatin combination chemotherapy only, without the addition of a monoclonal antibody. While the overall response rate in codon 13 patients was significantly lower, PFS was not different in the three *KRAS* mutational groups. Interestingly, we found a substantial difference in PFS between patients with codon 12 and 13 mutant tumors when looking at infusional 5-FU versus capecitabine based regimens. Patients with codon 13 mutations seem to benefit more in terms of PFS from the oral capecitabine based protocols. Moreover, there was a strong trend towards better overall survival in patients with wild type *KRAS* compared to all mutant *KRAS* patients. Lastly, when analysing OS in patients who received second and further line therapy we found that *KRAS* wild type and *KRAS* mutant patients alike showed a significantly higher OS post progression when treated with cetuximab.

A number of studies have looked at the potential prognostic or predictive value of *KRAS* mutations on response rates and survival in patients with CRC and several studies found a negative impact of *KRAS* mutations on prognosis [3,7,15-17].

Recently, a number of randomized trials have included translational research programs to evaluate certain target genes and their role as prognostic or predictive markers in patients with metastatic disease. Thereby, the mutational status of *KRAS* has now been established as a strong predictive marker of resistance to anti-EGFR-antibody treatment [5,8,10], although some trials could not fully confirm these results [4,11]. We still do not exactly know whether *KRAS* mutations influence the response to other treatment regimens such as standard chemotherapy or bevacizumab combinations. While bevacizumab efficacy seems independent from the *KRAS* status [18], the activity of certain chemotherapeutic agents may be influenced by *KRAS* mutations. There, patients seem to do worse under oxaliplatin combinations when carrying a mutant *KRAS* gene within their primary cancer [4,5,7,16,19,20]. For example, in the recently reported COIN study patients under oxaliplatin/5-FU combinations showed a median PFS of 8.6 months in the wild type *KRAS* cohort while median PFS was only 6.9 months in the *KRAS* mutant cohort [4]. Similarily, ORR was lower in *KRAS* mutant patients receiving chemotherapy with oxaliplatin only (41% vs. 50%). Furthermore, another recent small study evaluated the *KRAS* status in 66 patients receiving a second line chemotherapy with oxaliplatin and infusional 5-FU refractory to 5-FU/irinotecan based chemotherapy [16]. This study found a significantly lower response rate (7% vs. 27%, p = 0.026) and significantly shorter median PFS (3.1 vs. 5.2 months, p = 0.007) for patients with mutant *KRAS* tumors compared to patients with wild type *KRAS* tumors under oxaliplatin containing therapy.

Very recently, experimental and some clinical reports suggested that not all *KRAS* mutations behave alike [12,13,21]. In fact, there is evidence that patients carrying mutations in codon 13 of the *KRAS* oncogene which is found in about 8% of patients with advanced CRC have a substantially worse overall prognosis but may, on the other hand, benefit from anti-EGFR-treatment.

In our study we found a similar rate of codon 13 mutations as described before. The overall response rate in patients with codon 13 mutations in our analysis was as low as 23%, significantly lower than in patients with wild type or codon 12 mutations. The CRYSTAL- and the OPUS-studies alike found low response rates in patients with codon 13 mutations treated with combination chemotherapy only (17% for irinotecan combinations and 33% for oxaliplatin combinations) [13]. In contrast to previous reports, ORR was substantially higher in this codon 13 mutant patient cohort when cetuximab was added.

Our study was conducted using combination chemotherapy with oxaliplatin in first line treatment without the addition of monoclonal antibodies. Interestingly, the PFS and OS in our codon 13 cohort compared to the other mutated groups and other previously published works was rather long with a PFS of 10 months and an OS of 16.5 months [13]. We do not know why PFS and OS of the codon 13 cohort in our study was prolonged when response rates were as low as 23%. Either low patient numbers or a yet unknown functional mechanism of codon 13 mutated KRAS proteins within colon cancers may be responsible for the observation.

The present analysis also suggests that there may be an interaction between the type of *KRAS* mutation and the mode of application of 5-FU, i.e. whether administered intravenously or orally as capecitabine. In particular, patients with codon 13 mutations showed longer median PFS intervals when receiving capecitabine compared to infusional 5-FU. Although the overall efficacy of infusional 5-FU and capecitabine in advanced CRC has been found to be comparable [22] there may be some patient subpopulations or treatment regimen where the mode of application of the fluoropyrimidine is more crucial. For example, anti-EGFR antibodies in wild type *KRAS* patients may only be active when infusional 5-FU regimens are used, but not when capecitabine based protocols are applied [4].

The potential resistance of *KRAS* mutated tumors to oxaliplatin containing regimens seems interesting and may reveal more general mechanisms of drug resistance in cancers. Oxaliplatin belongs to the platinum containing compounds like cisplatinum and carboplatinum. Metabolites of platinum compounds interact with DNA and form crosslinks. In addition, platinum-DNA-adducts strongly inhibit DNA polymerases and therefore act antineoplastic. Some authors have studied in cell culture systems and preclinical models the influence of oncogenic RAS mutations on the activity of platinum compounds and found that the nucleotide excision repair protein ERCC-1 may be upregulated through activated RAS. ERCC-1 may subsequently activate DNA repair capacity and thus mediate platinum resistance. A recent study evaluated the role of ERCC-1 mRNA levels in 191 patients treated with FOLFOX within the CONFIRM1 and CONFIRM2 studies. Low ERCC-1 gene expression was correlated with higher response rates to FOLFOX chemotherapy and better overall survival. In contrast patients with high ERCC-1 did not have benefit from FOLFOX chemotherapy [23]. Of note, we have examined the expression of ERCC-1 by immunohistochemistry in our cohort and found no obvious correlation with *KRAS* status, response rates or survival and ERCC-1 expression (data not shown). The analysis of the ERCC-1 expression levels by RT-PCR as reported by other studies has not been performed so far [24].

Median overall survival after first progression correlated with cetuximab-treatment in patients bearing *KRAS* wild type and mutant tumors alike. The role of codon 13 mutation in this setting appears minor since there were only two patients with a codon 13 mutated tumor in the cohort with *KRAS* mutations of whom we know about cetuximab application. However, there are at least two limitations to our analysis. First, numbers are low in particular in the cetuximab group and secondly addition of cetuximab was not randomized for. There seems to be a selection of patients with good performance status and good prognosis who received third line therapy compared to those who did not. Therefore, we can not draw definite conclusions from our analysis whether anti-EGFR antibodies are effective in patients with *KRAS* G13D mutations or not.

We presently can not draw final conclusions regarding patient management from this study. It remains unclear whether chemotherapy backbones with irinotecan are less prone to interactions with mutated *KRAS* because data regarding this issue are conflicting. Independent validation of the findings is essential. Therefore, the standardized, thorough and comprehensive collection of tissue and blood samples of all trial patients within independent cancer tissue banks should be a major goal of modern clinical cancer trials.

## Misc

Anke Reinacher-Schick and Karsten Schulmann are contributed equally

## Competing interests

D. Arnold: Honoraria from Roche, Sanofi-Aventis. U. Graeven: Honoraria from Roche, Amgen, Sanofi-Aventis, Merck-Serono; Advisory Board Roche, Amgen. A. Reinacher-Schick: Honoraria from Amgen, Roche, Pfizer, Sanofi-Aventis; Advisory board member: Amgen, Roche, Pfizer; Studies sponsored by: Roche, Sanofi-Aventis. W. Schmiegel: Honoraria from Merck-Serono, Roche, Abott, Amgen, Astra-Zeneca, Pfizer, Falk; Advisory board member: Astra-Zeneca, Roche, Amgen; Studies sponsored by: Novartis, Amgen, Roche. K. Schulmann: Honoraria from Astra-Zeneca, Amgen, Falk; Travel support: Pfizer, Merck-Serono, Novartis. A. Tannapfel: Honoraria from Amgen, Roche, Pfizer, Sanofi-Aventis. Studies sponsored by: Roche, Sanofi-Aventis. The other authors declare that they have no competing interests.

## Authors’ contributions

ARS and KS: analysis and interpretation of data, drafting of the manuscript, revising the manuscript critically, final approval of manuscript. DPM: interpretation of data, biostatistic analysis, revising the manuscript critically, final approval of manuscript. NB: acquisition of data, analysis and interpretation of data, final approval of manuscript. UG: conception and design, recruitment of patients, revising the manuscript critically, final approval of manuscript. MJ: acquisition of data, analysis and interpretation of data, final approval of manuscript. RG: recruitment of patients, revising the manuscript critically, final approval of manuscript. RP and WS: recruitment of patients, conception and design, revising the manuscript critically, final approval of manuscript. DA: conception and design, revising the manuscript critically, final approval of manuscript. AT: conception and design, data acquisition, analysis and interpretation of data, revising the manuscript critically, final approval of manuscript. All authors read and approved the final manuscript.

## Pre-publication history

The pre-publication history for this paper can be accessed here:

http://www.biomedcentral.com/1471-2407/12/349/prepub
